# Knockdown of TACC3 inhibits tumor cell proliferation and increases chemosensitivity in pancreatic cancer

**DOI:** 10.1038/s41419-023-06313-x

**Published:** 2023-11-27

**Authors:** Saimeng Shi, Duancheng Guo, Longyun Ye, Tianjiao Li, Qinglin Fei, Mengxiong Lin, Xianjun Yu, Kaizhou Jin, Weiding Wu

**Affiliations:** 1 Department of Pancreatic Surgery, Fudan University Shanghai Cancer Center, Shanghai, 200032 China; 2 Department of Oncology, Shanghai Medical College, Fudan University, Shanghai, 200032 China; 3 Shanghai Pancreatic Cancer Institute, Shanghai, 200032 China; 4 Pancreatic Cancer Institute, Fudan University, Shanghai, 200032 China

**Keywords:** Pancreatic cancer, Mitotic spindle

## Abstract

Pancreatic ductal adenocarcinoma (PDAC) is a highly malignant digestive tract tumor with limited clinical treatments. Transforming acidic coiled-coil-containing protein 3 (TACC3) is a component of the centrosome axis and a member of the TACC family, which affect mitosis and regulate chromosome stability and are involved in tumor development and progression. However, the role of TACC3 in PDAC remains elusive. In this study, by exploiting the TCGA database, we found that high TACC3 expression in PDAC is associated with poor prognosis. shRNA-mediated TACC3 knockdown caused S phase arrest of the cell cycle and inhibited proliferation in PDAC cell lines. Through RNA sequencing and protein co-immunoprecipitation combined with mass spectrometry, KIF11 was identified as a protein that interacts with TACC3. TACC3 stabilizes and regulates KIF11 protein expression levels in PDAC cells through physical interaction. Knockdown of TACC3 or KIF11 resulted in abnormal spindle formation during cell division both in vitro and in vivo. Pharmacological inhibition of TACC3 or KIF11 can suppress tumor cell proliferation and promote apoptosis. Our studies further demonstrated that high expression of TACC3 and KIF11 mediated the resistance of PDAC to gemcitabine, and deficiency of TACC3 or KIF11 increased the sensitivity of PDAC cells to chemotherapy. In conclusion, our study reveals the fundamental role of TACC3 expression in PDAC cell proliferation and chemoresistance, suggesting that TACC3 can be used as a molecular marker to evaluate the prognosis of PDAC.

## Introduction

Pancreatic ductal adenocarcinoma (PDAC) is a highly lethal digestive malignancy with an overall 5-year survival rate of only 10% [1]. In recent years, the incidence of PDAC has been increasing worldwide, and PDAC has now become the third leading cause of cancer death worldwide [2]. Surgery is still the only radical treatment for PDAC patients, but the vast majority of patients have already lost the opportunity for surgery due to disease progression by the time the tumor is first detected. Neoadjuvant chemotherapy may offer hope for resection in some of these patients, but more effective and evidence-based treatment options are needed [3, 4]. Similarly, although they are promising treatments, targeted therapies and immunotherapies have not made sufficient progress [5].

Transforming acidic coiled-coil-containing protein 3 (TACC3), a member of the TACC family, encodes a motor spindle protein characterized by a highly conserved C-terminal coiled-coil domain called the “TACC domain”. During mammalian cell division, the assembly of the bipolar spindle based on the microtubule structure is one of the key steps to ensure that the duplicated chromosomes are precisely separated into the two daughter cells and that the genetic material is evenly distributed [6, 7]. TACC3 is believed to play an important role in controlling microtubule nucleation and stability [8]. During both meiosis in human oocytes and mitosis in mammalian somatic cells, TACC3 participates in the formation and assembly of the bipolar spindle [9–11]. High expression of TACC3 may be associated with some human cancers. In various cancers, including breast cancer, lung cancer, prostate cancer, and adult leukemia/lymphoma, TACC3 presents abnormal expression, and high TACC3 expression is usually associated with a poor prognosis [12–16]. TACC3 is currently an important molecular marker for cancer diagnosis and prognostic prediction, but the specific mechanism and role of TACC3 in tumorigenesis remain to be further elucidated. In recent years, TACC3 has also been found to be highly expressed in PDAC. Wang et al. reported a lncRNA named pancreatic cancer TACC-3 suppressive transcript (lnc-PCTST) with aberrant down-regulated expression in pancreatic cancer tissues [17]. In patients with pancreatic cancer, low lnc-PCTST expression was associated with adverse clinicopathological features, including larger tumor volume, advanced TNM stage, shorter overall survival, and increased cancer-related death risk. Lnc-PCTST inhibited the proliferation and invasion of pancreatic cancer cells in vitro and hindered tumorigenesis in vivo. Further mechanism study showed that lnc-PCTST significantly inhibited the activity of TACC3 promoter. That is, abnormally low expression of Lnc-PCTST in pancreatic cancer leads to high expression of TACC3, which promotes the proliferation and invasion ability of cancer cells. The study by Wang et al. alluded a cancer-promoting role of TACC3 in pancreatic cancer, however, its specific functions and mechanism has not been further explored.

Our current study showed that TACC3 was highly expressed in PDAC and exhibited a clear positive correlation with poor prognosis. Through a series of functional tests and in vivo experiments, we confirmed that TACC3 supports the malignant phenotype of PDAC and participates in regulation of the cell cycle. Knockdown of TACC3 leads to abnormal spindle formation and S phase arrest during PDAC cell division, which inhibits tumor cell proliferation. Through a comprehensive analysis of RNA sequencing, co-immunoprecipitation (Co-IP), and protein-protein interaction (PPI) network resources, we identified the downstream protein that interacts with and is regulated and stabilized by TACC3 as KIF11, a motor protein associated with spindle dynamics. KIF11 supports and promotes proliferation and spindle formation in PDAC cells. Moreover, the TACC3 inhibitor KHS101 inhibited the progression of PDAC in vivo. Finally, we demonstrated that knockdown of TACC3 sensitizes PDAC cells to chemotherapy drugs. In conclusion, our study reveals a key role of TACC3 in mitotic progression and the malignant phenotype in PDAC cells and shows that targeting TACC3 may provide a promising treatment for PDAC.

## Materials and methods

### Patients and specimens

Clinical tissue samples were obtained from patients underwent radical surgery for pancreatic cancer at Fudan University Shanghai Cancer Center (FUSCC) from November 2011 to May 2014 and were diagnosed as PDAC pathologically and clinically. All patients met strict inclusion criteria, and the following patients were excluded: (1) tumor involved the celiac axis, superior mesenteric artery, or common hepatic artery; tumor involved superior mesenteric vein or portal vein; (2) tumor developed distant metastasis (3) underwent preoperative chemotherapy; (4) died due to postoperative complications. Tumor resectability complied with the latest NCCN guidelines and all enrolled patients achieved R0 resection. The clinical tissue samples of patients with chemotherapy were obtained from patients who received preoperative neoadjuvant chemotherapy and subsequent radical surgery for pancreatic cancer at FUSCC between October 2015 to August 2019 and were diagnosed as PDAC pathologically and clinically. The tumors were localized and had not been previously treated. These patients received at least 3 cycles of chemotherapy prior to surgery, with a chemotherapy regimen of gemcitabine combined with albumin-paclitaxel.

### Cell culture

HEK293T cells, H6C7 cells and human pancreatic cancer cell lines (SW1990, BxPC-3, Mia-PaCa-2, Capan-1, CFPAC-1, and Panc-1) were purchased from Cell Bank, Type Culture Collection, Chinese Academy of Sciences. All human cell lines have been authenticated using STR profiling and tested with no mycoplasma contamination. The cells were cultured in a humidified incubator with 37 °C, 5% carbon dioxide. H6C7 and BxPC-3 were cultured in RPMI-1640 with 10% FBS; Capan-1 and CFPAC-1 were cultured in IMDM with 10% FBS; and other cells were cultured in high glucose DMEM with 10% FBS.

### Lentivirus package and Tet-On system

Lentiviral plasmids expressing TACC3 or KIF11 were produced by Tsingke Biotechnology. To generate lentiviral particles, HEK293T cells at 60%-70% confluence in a 100 mm dish were co-transfected with 3ug target plasmid, 9ug psPAX2, 9ug pMD2.G using Lipofectamine™ 3000 (L3000075, Thermo Fisher, Carlsbad, CA, USA) as a gene delivery carrier. Cells were refreshed with 15 mL full medium after incubated at 37 °C for 6 h and lentiviral particle-enriched supernatant was collected 48 h after virus packaging. Using these lentiviral particles to infect Panc-1 cells and the stable expressing cells were established with 10 μg/ml puromycin for 1–2 weeks.

shRNA is used to knockdown the expression of TACC3 or KIF11 in Panc-1 cells. The sequences for shRNA are below:

sh-Scr: CCTAAGGTTAAGTCGCCCTCG;

shTACC3#1: GCTTGTGGAGTTCGATTTCTT;

shTACC3#2: GTTACCGGAAGATCGTCTGT;

TACC3-3’UTR: CCACGGAGCCGCTGTCCCCGC;

shKIF11#1: GGAGGAGATAGAACGTTTAAA;

shKIF11#2: CACGTACCCTTCATCAAATTT.

Doxycycline (DOX)-inducible Tet-On system was constructed according to the standard procedure: Briefly, the gene sequences of sh-Scr, shTACC3#1, shTACC3#2 were introduced into the DOX induced expression vector by PCR, transformed in by E. coli, and then monoclonal colonies were picked and sent for sequencing. Lentivirus was packaged and Panc-1 cells were infected with lentivirus to obtain stable strains that express the corresponding gene under 2 μg/mL DOX induction for 48 h.

### Animal model and drug treatment experiment

All mice were purchased through the Laboratory Animal Center of FUSCC and bred in the specific pathogen-free facility. *LSL-Kras*^G12D/+^*LSL-Trp*53^R172H/+^; *Pdx-1-Cre* mice were purchased from Gempharmatech Co., Ltd, Jiangsu, China. and genotyped following the company’s recommended standard protocol. *LSL-Kras*^G12D/+^*LSL-Trp53*^R172H/+^*Pdx-1-Cre* (KPC) transgenic mice developed PDAC after 8 weeks of age. Subcutaneous tumor model was established to use in basic research. Briefly, female BALB/c-nu mice, aged 4–6 weeks, were subcutaneously injected with about 5 × 10^6^ Panc-1 cells in 100 μL PBS. Tumor volume was measured and calculated = (width^2^×length)/2. When the tumor volume reached ~200 mm^3^, the mice were randomly grouped and received the corresponding treatment. For Tet-On system, each group of mice were fed with PBS containing 2 μg/mL DOX to regulate the gene expression. For TACC3 inhibitor treatment experiments, KHS101 (T4968, TOPSCIENCE, Shanghai, China) was dissolved in 3% DMSO in MCT (0.5% methyl cellulose containing 0.2% Tween-80, Solarbio, Beijing, China). The tumor-bearing mice were given intraperitoneal injections of 15 mg/kg, 30 mg/kg KHS101, or vehicle control. Treatments for both experiments were performed once a day for 2 weeks. For the combination therapy experiment, each group of mice were fed with PBS containing 2 μg/mL DOX to regulate the TACC3 expression, combined with an intraperitoneal injection of 200 μL PBS or gemcitabine (20 mg/kg) once every 2 days. No blinding method was used for injection. There were no animal exclusion criteria. Tumor volume was measured every 2 days. At the end of the treatment, tumors were collected and processed for sections, RNA and protein samples prepared. All animal experiments were conducted in accordance with the Institutional Animal Care and Use Committee of FUSCC.

### Tissue microarray (TMA), immunohistochemistry (IHC) and immunofluorescent staining (IF)

The tissue samples were fixed with 4% paraformaldehyde and embedded in paraffin, and then were sent to Wuhan Servicebio Technology for TMA fabrication and IHC staining. Hematoxylin-eosin (H&E) staining was used for histological examination. Antibodies used for IHC include anti-TACC3 (1:100; 25697-1-AP, Proteintech, Wuhan, China); anti-Ki-67 (1:200; 27309-1-AP, Proteintech); anti-KIF11 (1:100; 23333-1-AP, Proteintech).

Pathological diagnosis and IHC scoring were performed independently by two pathologists blinded to the clinical characteristics. Five 20× fields were selected randomly for each section and 100 cells were counted in each field. The proportion of stained cells was divided into 5 grades as following criteria: 0 ( <5%), 1 (6–25%), 2 (26–50%), 3 (51–75%) and 4 ( >75%). The intensity of immunostaining was divided into 4 grades as following criteria: 0 (negative), 1 (weak), 2 (moderate), and 3 (strong). IHC score was obtained by multiplying the positive rate and the staining intensity [18]. A score between 0 and 6 is classified as low expression, and 7 to 12 is high expression.

IF staining: The previous animal tissue samples were frozen and cut into slices of 10 mm thickness according to the standard method. Sections were blocked and permeabilized with PBS containing 1% BSA and 0.2% Triton X-100 for 1 h at room temperature, incubated with primary antibody at 4 °C overnight and then incubated with fluorescein-conjugated secondary antibody for 2 h at room temperature. Nuclei were counterstained with DAPI for 10 min. Finally, the slides were mounted with Fluoromount-G and observed under a Zeiss microscope. IF staining procedure of cells was the same as that described above. Before staining, cells were fixed with 4% paraformaldehyde and permeabilized with 0.2% Triton X-100. Primary antibodies used for IF included anti-TACC3 (1:400; 25697-1-AP, Proteintech); anti-Ki-67 (1:200; 27309-1-AP, Proteintech); anti-Cleaved-caspase 3 (CC3, 1:1000; 9664 S, Cell Signaling Technology, CST); anti-α-tubulin (1:200; 66031-1-Ig, Proteintech); anti-KIF11 (1:500; 23333-1-AP, Proteintech). The secondary antibodies were: goat anti-mouse CoraLite594 (1:200; Proteintech, SA00013-3) and goat anti-rabbit CoraLite594 (1:200; Proteintech, SA00013-4).

### Western blotting (WB), co-immunoprecipitation (Co-IP) and mass spectrometry (MS)

Total tissue or cellular proteins were extracted from RIPA buffer containing protease inhibitor and phosphatase inhibitor cocktail, separated by SDS-PAGE gels and then transferred to a PVDF membrane. The membrane was incubated with the primary antibody at 4 °C overnight and incubated with the horseradish peroxidase-conjugated second antibody at room temperature for 2 h. Finally, the bands were displayed with high signal ECL substrate (Beyotime, Shanghai, China), and protein bands were visualized Clinx Chemiluminescence Instrument. Primary antibodies used for WB included anti-GAPDH (1:50000; 60004-1-Ig, Proteintech); anti-TACC3 (1:1000; 25697-1-AP, Proteintech); anti-KIF11 (1:2000; 23333-1-AP, Proteintech); anti-RRM2 (1:5000; ab172476, Abcam, Cambridge, CB2 0AX, UK); anti-CDKN1A (1:2000; 10355-1-AP, Proteintech); anti-Caspase-3 (1:1000; 9662, CST); anti-Cleaved-caspase 3 (1:1000; 9664 S, CST). The secondary antibodies were goat anti-rabbit IgG secondary antibody HRP conjugated (1:5000; L3012, Signalway Antibody, Greenbelt, MD, USA); goat anti-mouse IgG secondary antibody HRP conjugated (1:5000; L3032, Signalway Antibody).

Total proteins were extracted from the samples, part of which were used to perform Co-IP according to the manufacturer’s instructions (Cytoskeleton, Denver, CO, USA), and negative controls were performed using the same concentration of a subclass-matched immunoglobulin. Another part of the proteins was sent to Shanghai Oebiotech for MS and data analysis.

### Conventional PCR and real-time quantitative PCR (qPCR)

Total RNA was extracted from tissues or cells using a RNAeasy™ Animal RNA Isolation Kit with Spin Column (R0027, Beyotime) and the RNA purity was detected with a NanoDrop One spectrophotometer (Thermo Fisher). cDNA was synthesized using 1st Strand cDNA Synthesis Kit (R312-01, Vazyme, Nanjing, China). Conventional PCR was performed following standard protocols and visualized by agarose gel. qPCR was performed in triplicate using SYBR qPCR Master Mix (Q711-02, Vazyme) and Applied Biosystems QuantStudio 6 Real-Time PCR Detection System (Thermo Fisher). The differences in mRNA expression were calculated by the 2^−ΔΔCt^ method. Sequences of the primers used in this study are as follows (all for human species):

GAPDH (forward: 5′-GTCTCCTCTGACTTCAACAGCG-3′; reverse: 5′-ACCACCCTGTTGCTGTAGCCAA-3′); TACC3 (forward: 5′-TCTTGGGAGCACTGGACATTCC-3′; reverse: 5′-TCCAGGTCCTTCTGGCTGTACT-3′); KF11 (forward: 5′-TACAGAAACCACTTAGTAGTGTCC-3′; reverse: 5′-GAGTTCCTGTGAGAAGCCATCAG-3′)

### EdU assay

Cell proliferation was measured by using a BeyoClick™ EdU-594 kit (Beyotime). Cells were seeded in 24-well plates at a density of 1×10^5^ cells per well in triplicate. When the cells proliferated for 48 h, EdU(10 μM) was added to each well and incubated for 2 h at 37 °C to label cells. Then the cells were fixed with 4% paraformaldehyde and permeated with 2%Triton X-100. Finally, Click Addictive Solution was added to cells and incubated for 30 min to develop color. Using a Zeiss microscope to observe.

### Transwell assay

Transwell filters (24-well, 8μm pore diameter, CLS3422, Corning, Corning, NY, USA) were placed in a 24-well plate, and the lower chamber was added with 400 μL full medium. 1×10^4^ Panc-1 cells were seeded in 200 μL serum-free medium in the Matrigel-free upper chamber. After 12 h of culture, the cells in the lower chamber were fixed with 4% paraformaldehyde, stained with Crystal Violet and counted in randomly selected fields.

### Cell cycle analysis

Cell cycle was measured according to the instructions of Cell Cycle and Apoptosis Analysis kit (C1052, Beyotime). Briefly, the cells were fixed with 70% ethanol, washed with PBS and stained with propidium iodide. Cell cycle analysis was performed by using a Flow Cytometry System (Beckman Coulter CytoFlex S) and CytExpert software (version 2.3.1.22). Each analysis collected at least 20,000 cells.

### CCK-8 assay

Cell viability and proliferation were measured by Cell Counting Kit-8 (CCK-8, B34302, Selleck, Houston, TX, USA). Cells were seeded in 96-well plates at a density of 1×10^4^ cells per well in triplicate, and treated with chemotherapy drugs such as gemcitabine, paclitaxel, or cisplatin for 48 h before being harvested to measure their viability. Then CCK-8 reagent was added to each well and incubated at 37 °C for 2 h. The absorbance at 450 nm was measured with a microplate reader. Each assay was performed in triplicate.

### RNA sequencing

Cell samples were lysed with TRIzol reagent (15596018, Thermo Fisher Scientific) and total RNA was extracted according to the standard protocol. Then, RNA sequencing was performed (Novogene Co., Ltd, Beijing, China) and sequencing results were analyzed by using R-4.1.3 software.

### Statistical analysis

All the experiments in this study were performed in at least triplicate. Data were analyzed by using GraphPad Prism 9 software and presented as the mean ± standard of measurement. Continuous variables were compared by using the *Student’s t*-test. Categorical variables were compared by using Chi-square (or the Fisher exact test when appropriate). Survival data were analyzed by using Kaplan-Meier survival curves and Log-rank test. *P* < 0.05 was considered statistically significant. No statistical methods were used to predetermine the sample size. The variance was similar between the groups that were being statistically compared. Data were graphically display by using the GraphPad Prism9. Bioinformatics analysis was performed by using R (3.1.4) software.

## Results

### TACC3 is highly expressed in PDAC and its expression is inversely correlated with patient survival

**Fig. 1:**
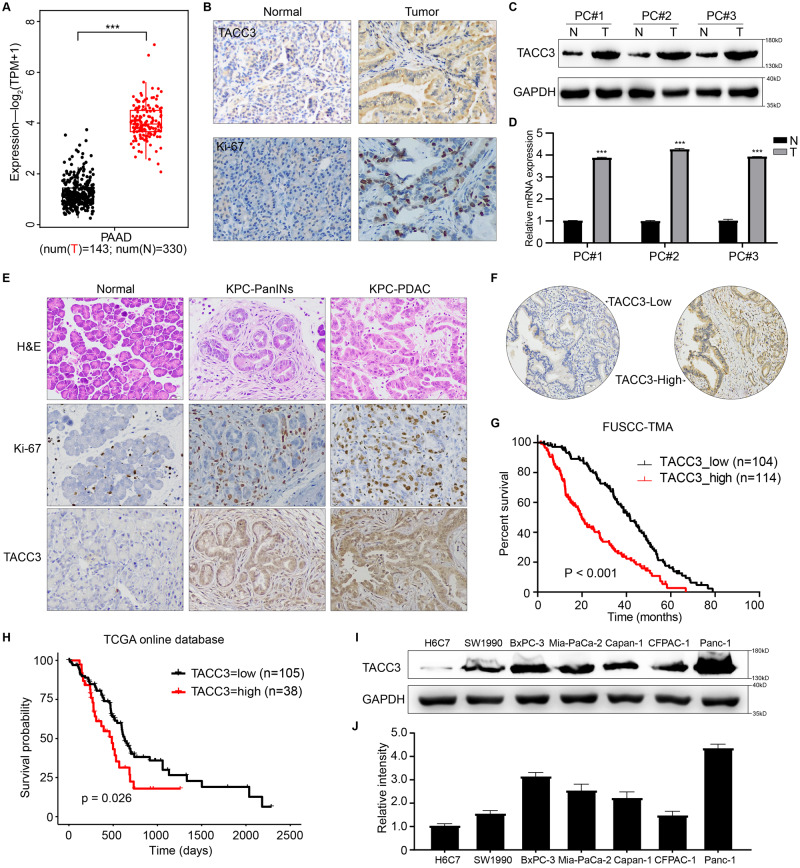
TACC3 is highly expressed in PDAC and inversely correlates with patient survival. **A** Differential expression of TACC3 between different disease states (Tumor or Normal) in PDAC was analyzed by TCGA and GTEx database. **B** Representative images of negative TACC3 IHC staining in normal pancreas and representative images of strong positive TACC3 IHC staining in pancreatic ductal adenocarcinoma (PDAC) tissue; Representative images of weakly positive Ki-67 IHC staining in normal pancreas and representative images of strongly positive Ki-67 IHC staining in PDAC tissue. Scale bar: 100 µm. **C** Western blotting analysis of TACC3 expression levels in three paired PDAC tissues (T) and adjacent normal pancreatic tissue (N). **D** qPCR analysis of TACC3 mRNA expression levels in three paired PDAC tissues (T) and adjacent normal pancreatic tissue (N). 10 PDAC tissue samples from *LSL-Kras*^G12D/+^*LSL-Trp53*^R172H/+^*Pdx-1-Cre* (KPC) mice and 10 normal pancreatic tissue samples from wild-type (WT) C57BL/6 J mice were collected for H&E staining and IHC staining analysis. **E** Representative images of H&E staining, Ki-67 IHC staining, TACC3 IHC staining of normal pancreatic tissue, pancreatic intraepithelial neoplasia (PanINs) and PDAC tissue. Scale bar: 50 µm. **F** Representative images of high/low TACC3 protein expression in PDAC samples from FUSCC-TMA samples. Scale bar: 200 µm. **G** In PDAC tissue samples from TMA, Kaplan-Meier survival analysis was used to assess the association between high/low expression of TACC3 and clinical outcome. High TACC3 expression was significantly associated with poor prognosis (median: 19.73 months vs. 41.50 months, respectively; log-rank test, *P* < 0.001). **H** Clinical samples acquired from TCGA and analyzed by R-4.1.3 showed that high TACC3 expression was associated with poor prognosis in PDAC (median: 485 days vs. 627 days, respectively; log-rank test, *P* = 0.026). **I** Western blotting examined the abundance of TACC3 in six established human pancreatic cancer cell lines, using H6C7 human normal pancreatic duct epithelial cells as a control; using GAPDH as a loading control. **J** qPCR examined the abundance of TACC3 in six established human pancreatic cancer cell lines, using H6C7 human normal pancreatic duct epithelial cells as a control. (All studies were performed with three biological replicates).

In combination with the TCGA and GTEx database, we compared the mRNA expression level of TACC3 in 143 PDAC tissues and 330 normal pancreatic tissues. TACC3 expression was markedly up-regulated in PDAC tissues (Fig. 1A). Next, we examined the abundance of the TACC3 protein via IHC staining of 46 pairs of PDAC and adjacent normal pancreatic tissues from our center. We found that TACC3 was weakly expressed in normal pancreatic tissue but strongly expressed in PDAC tissue (Fig. 1B). Meanwhile, IHC staining of Ki-67 protein showed that the cells in tumor tissues were in an aberrant state of vigorous proliferation [19]. In addition, consistent results about the differential expression of TACC3 were obtained by Western blotting and qPCR detection, as shown in Fig. 1C, D. Similarly, TACC3 levels were higher in tumor tissue from *LSL-Kras*^G12D/+^*LSL-Trp53*^R172H/+^*Pdx-1-Cre* (KPC) mice than in normal pancreatic tissue from wild-type C57BL/6 J mice (Fig. 1E). It is worth noting that there are also differences in TACC3 abundance at different stages of PDAC tissues in KPC mice as the disease progression. Compared with pancreatic intraepithelial neoplasia (PanINs), the TACC3 expression is relatively higher in advanced PDAC. Meanwhile, the proportion of proliferating (Ki-67^+^) cells was also greater in tumor tissue from KPC mice than in normal tissue from wild-type mice. These results indicated that TACC3 expression was significantly up-regulated in PDAC tissues, with its abundance increases as tumor progression.

To further investigate the prognostic value of TACC3 expression, we collected tumor tissues from 218 patients with PDAC at our center to perform a tissue microarray (TMA). By performing IHC staining, we evaluated TACC3 abundance and quantified expression differences. Patients were divided into a high-TACC3 expression cohort (*n* = 114) and a low-TACC3 expression cohort (*n* = 104) based on TACC3 expression by IHC staining (Fig. 1F). High TACC3 expression was significantly associated with poor prognosis (median: 19.73 months vs. 41.5 months for high TACC3 expression vs. low TACC3 expression, respectively; log-rank test, *P* < 0.001; Fig. 1G). Similarly, after downloading and analyzing the 143 clinical samples of PDAC from the TCGA database, consistent conclusions were drawn (median: 485 days vs. 627 days for high TACC3 expression vs. low TACC3 expression, respectively; log-rank test, *P* = 0.027; Fig. 1H). Data from the above tissue sample bank and online database suggest that high expression of TACC3 in PDAC is associated with poor patient prognosis. Subsequently, we analyzed the correlations between TACC3 expression and PDAC clinicopathologic parameters in 218 patients (Supplementary Table 1). The results showed that high TACC3 abundance correlated positively with more extensive neural invasion, more severe microvascular invasion, more advanced 8^th^ AJCC stage, poorer tumor differentiation, larger tumor volume, increased lymph node metastasis, higher serum CA199 level and greater tumor cell proliferation capacity (Table 1). Taken together, these results show that TACC3 is significantly highly expressed in PDAC and clearly associated with poor prognosis. Therefore, TACC3 may play an important role in the development and progression of PDAC.

**Table 1:** Relationship between TACC3 expression and clinicopathological features of patients with PDAC.

Characteristics	The Pooled(*N* = 218)	TACC3		*P*-value
		Low (*N* = 104)	High (*N* = 114)	
Age(years)				0.986
≤60	90	43	47	
<60	128	61	67	
Gender				0.976
Male	126	60	66	
Female	92	44	48	
Tumor location				0.477
Head and neck	127	58	69	
Body and tail	91	46	45	
Neural invasion				0.039
No	42	26	16	
Yes	172	76	96	
Microvascular invasion				0.013
No	149	80	69	
Yes	57	20	37	
Tumor differentiation				0.029
Poor	75	32	43	
Moderate	106	47	59	
Well	37	25	12	
8^th^ AJCC stage				0.005
I	89	52	37	
II	103	46	57	
III	26	6	20	
8^th^ T stage				0.012
I	28	20	8	
II	124	59	65	
III	66	25	41	
8^th^ N stage				0.027
0	121	67	54	
1	68	28	40	
2	29	9	20	
Preoperative CA19-9 (U/ml)				0.042
≤37	48	29	19	
<37	169	74	95	
Ki-67				<0.001
Low	93	57	36	
High	77	22	55	

To select suitable cell lines for in vitro experiments, we evaluated the abundance of TACC3 in established human pancreatic cancer cell lines, including the SW1990, BxPC-3, Mia-PaCa-2, Capan-1, CFPAC-1, and Panc-1 cell lines, by Western blotting and qPCR. H6C7 human normal pancreatic duct epithelial cells were used as controls. As shown in Fig. 1I, J, TACC3 expression varied across pancreatic cancer cell lines, with the highest expression in Panc-1 cells. Therefore, we used Panc-1 cells, which had the highest TACC3 expression, to investigate the function of TACC3 in pancreatic cancer cells.

### TACC3 supports the malignant phenotype of PDAC cells and participates in regulation of the cell cycle

**Fig. 2:**
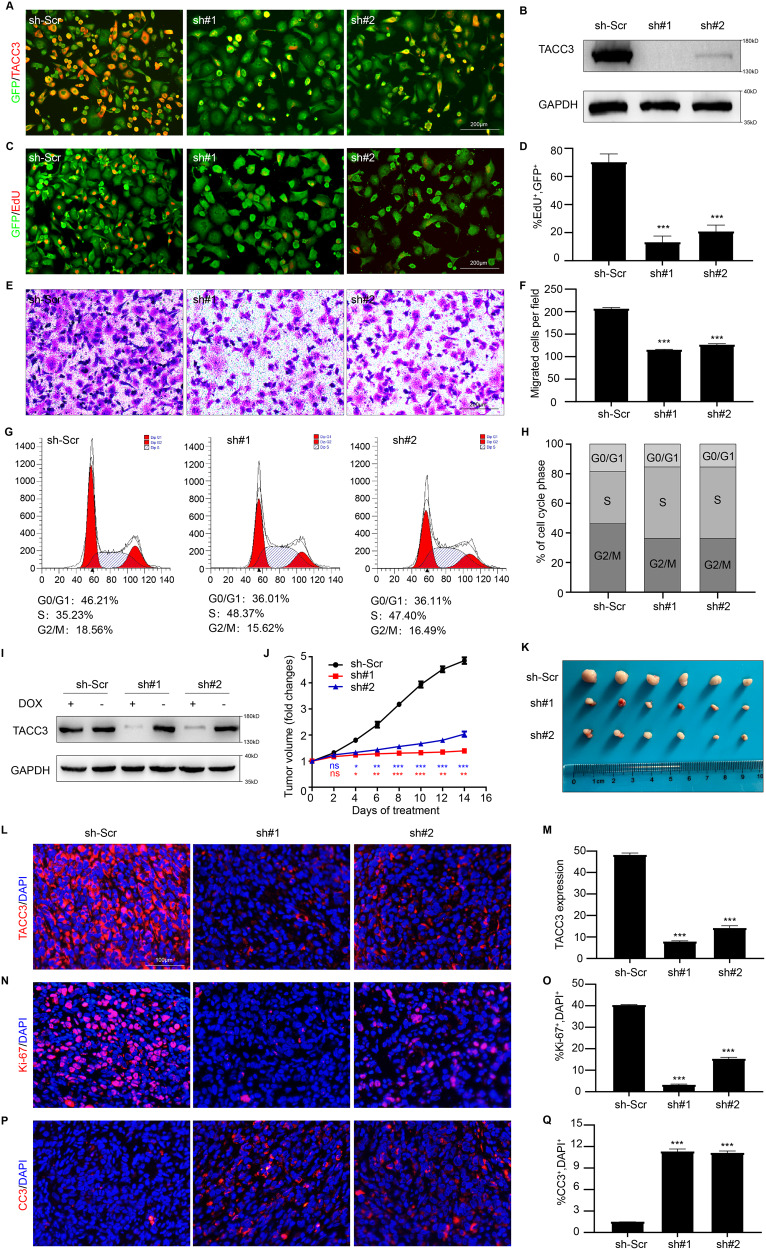
TACC3 supports the malignant phenotype of PDAC cells and participates in the regulation of cell cycle. **A**–**H** Panc-1 cells were infected with a scrambled shRNA (sh-Scr) or TACC3 shRNA (sh#1, sh#2). **A** IF staining of TACC3 protein (in red color) in sh-Scr/sh#1/sh#2 Panc-1 cells (GFP^+^, in green color) to verify transfection efficiency. Scale bar: 200 µm. **B** Western blotting was used to evaluate TACC3 protein levels in control cells and TACC3-deficient cells to verify transfection efficiency; GAPDH was used as a loading control. **C** Representative images of EdU assays detecting the proliferation of control cells and TACC3-deficient cells, and (**D**) the percentage of EdU^+^ cells (in red color) per field was quantified. Scale bar: 200 µm. **E** Representative images of Transwell assays detecting the migration ability of control cells and TACC3-deficient cells, and (**F**) the number of migrated cells per field was counted. Scale bar: 200 µm. **G** Flow cytometry of cell-cycle distribution of control cells and TACC3-deficient cells, and (**H**) visualized with a stacked percentage bar plot. **I**–**Q** A subcutaneous PDAC model of Panc-1 cells was constructed to explore TACC3 functions in vivo, and TACC3 expression was regulated by the DOX-inducible Tet-on system. *n* = 6 biologically independent mice per group. **I** Western blotting validated the efficiency of DOX-induced TACC3 knockdown in Panc-1 cell line. **J** Volume changes of subcutaneous tumor was monitored every 2 days and displayed by line charts. **K** On the 14th day of DOX treatment, tumors were separated and photographed to show tumor size. Frozen sections were made from subcutaneous tumor tissue. IF staining of TACC3 protein (in red color, (**L**)) was performed to detect its expression levels; and of Ki-67 (in red color, (**N**)) and CC3 (in red color, (**P**)) to show proliferation and apoptosis. Using DAPI to counterstain the cell nuclei (in blue color). Scale bar: 100 µm. The TACC3 expression intensity (**M**) Ki-67^+^ cell percentage (**O**) and CC3^+^ cell percentage (**Q**) per field were quantified. (Values are shown as the means ± SDs. *P < 0.05, **P < 0.01, ***P < 0.001. Unless otherwise specified, *n* = 3. All studies were performed with three biological replicates).

To investigate the possible role of TACC3 in PDAC cells, Panc-1 cells were infected with a lentivirus expressing TACC3-specific shRNA, and a scrambled shRNA was used as a control. Immunofluorescence (IF) staining results showed that infection with the TACC3 shRNA lentivirus effectively reduced TACC3 protein expression in Panc-1 cells (Fig. 2A). Western blotting also confirmed shRNA-induced suppression of TACC3 expression (Fig. 2B). Next, we compared the proliferative capacity of control cells (sh-Scr) and TACC3-deficient cells (sh#1 and sh#2). EdU assays showed that TACC3 knockdown significantly inhibited the growth of Panc-1 cells in vitro (Fig. 2C, D). In addition, the migration ability of Panc-1 cells decreased substantially after TACC3 knockdown, as shown by the Transwell assay results (Fig. 2E, F). These results suggest that TACC3 is essential for the proliferation and migration of PDAC.

The ability to maintain chronic proliferation is one of the most fundamental features of cancer cells [20]. In cancer cells, the signals that dynamically inhibit the cell growth and division cycle are deregulated, and progression through the cell cycle is significantly altered. We wondered whether TACC3 influences the proliferative characteristics of PDAC cells by regulating the cell cycle. To answer this question, we analyzed the differences in cell cycle distribution between control cells and TACC3- deficient cells. The results showed a significant increase in the proportion of cells in S phase after TACC3 knockdown (Fig. 2G, H). These results suggested that TACC3 deficiency may lead to arrest at the S-G2 transition in Panc-1 cells, thus weakening the proliferation ability of the cells.

To explore whether TACC3 also supports and promotes PDAC progression in vivo, we constructed a subcutaneous tumor model with Panc-1 cells in mice and regulated TACC3 gene expression via the doxycycline (DOX)-inducible Tet-On system [21, 22]. We verified the successful construction of the Tet-On system by Western blotting of samples from cell lines: the addition of DOX (2 μg/mL) induced a decrease in the TACC3 expression level (Fig. 2I). BALB/c-nu mice were randomly divided into 3 groups, and subcutaneous tumors were established using the above three cell lines (sh-Scr, sh#1, and sh#2). When the tumor volume reached ~200 mm^3^, each group of mice was fed daily with PBS containing 2 μg/mL DOX to induce expression of the corresponding genes. Tumor size was measured and recorded every 2 days (Fig. 2J). All tumor specimens were removed after 14 days of treatment, and we observed that the tumor progression of the TACC3-deficient group was significantly slower than that of the control group (Fig. 2K). IF analysis (Fig. 2L, M) of subcutaneous tumors confirmed the efficiency of DOX-induced TACC3 knockdown, which caused markedly attenuated proliferation (Fig. 2N, O) and enhanced apoptosis (Fig. 2P, Q) in Panc-1 cells. Collectively, the above in vitro and in vivo experiments demonstrated that TACC3 supports malignancy-related phenotypes, such as the proliferation and migration of PDAC cells, and is involved in the regulation of the cell cycle.

### TACC3 deficiency impairs spindle formation in PDAC cells

**Fig. 3:**
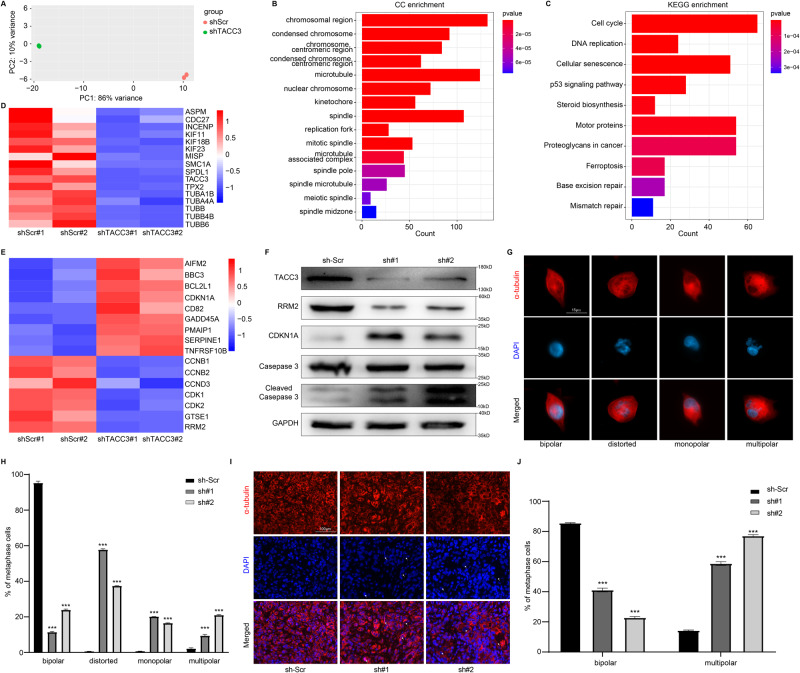
TACC3 deficiency impairs spindle formation in PDAC cells. **A**–**F** RNA sequencing was performed to examine the gene expression profiles of control Panc-1 cells and TACC3-deficient Panc-1 cells. *n* = 2 per group. **A** Principal component analysis (PCA) plot of the differentially expressed genes. **B** GO (cellular component) and (**C**) KEGG enrichment analysis of differentially expressed genes. **D** Cluster heatmap showing the expression of typical cell cycle-associated and spindle microtubule-associated proteins in control cells and TACC3-deficient cells. **E** Cluster heatmap showing the expression of typical p53 signaling pathway genes in control cells and TACC3-deficient cells. **F** Western blotting showing the expression of typical p53 signaling pathway protein in control cells and TACC3-deficient cells; GAPDH was used as a loading control. **G** Spindle morphology of control cells and TACC3-deficient cells was detected by IF staining with an anti- α-tubulin antibody (in red color). Nuclei were counterstained with DAPI (in blue color). Scale bar: 15 µm. **H** The percentage of Panc-1 cells with normal (bipolar) or abnormal spindles (including distorted, monopolar, or multipolar) was quantified. **I**, **J** Frozen sections prepared from the afore-mentioned subcutaneous tumor models was used to detect (**I**) spindle morphology by IF staining with an anti- α-tubulin antibody (in red color). Nuclei were counterstained with DAPI (in blue color). Scale bar: 100 µm. **J** The percentage of Panc-1 cells with bipolar or multipolar spindles was quantified. (Values are shown as the means ± SDs. *P < 0.05, ** *P* < 0.01, *** *P* < 0.001. Unless otherwise specified, *n* = 3. All studies were performed with three biological replicates).

To further explore the possible mechanism by which TACC3 regulates the cell cycle and promotes PDAC progression, we performed RNA sequencing to examine the gene expression profiles of Panc-1 cells transfected with scrambled shRNAs and TACC3 shRNAs (Supplementary Table 2). Principal component analysis (PCA) revealed the different gene expression profiles of these two groups of cells (Fig. 3A). By performing GO (Gene Ontology) enrichment analysis, we found that these differentially expressed genes were significantly enriched in cellular components associated with spindles/microtubules after TACC3 knockdown (Fig. 3B). In addition, enrichment analysis of KEGG (Kyoto Encyclopedia of Genes and Genomes) pathways showed that these differentially expressed genes were significantly enriched in pathways related to the cell cycle and motor proteins (Fig. 3C). These results are consistent with the established role of TACC3 as a motor spindle protein, suggesting that TACC3 may be involved in regulating spindle formation and assembly in PDAC, thereby affecting cell cycle regulation and proliferation. Therefore, we plotted a heatmap of the relative expression of some representative genes related to the spindle. The cluster heatmap showed that many cell cycle-associated proteins (such as CDC27) and spindle-associated proteins were consistently downregulated in TACC3-deficient cells (Fig. 3D). Importantly, TUBA1B, TUBA4A, TUBB, TUBB4B, and TUBB6, which are closely related to tubulin, a skeletal protein, were identified among these spindle-associated proteins [23, 24]. These results suggest that TACC3 might affect spindle assembly by influencing spindle/microtubule function and thus participate in the regulation of the cell cycle.

P53 signaling pathway was also highly enriched in KEGG enrichment analysis. Heatmap of the relative expression of some representative genes in p53 signaling pathway showed that the knockdown of TACC3 increased the expression of p53-induced genes (such as pro-apoptotic genes: *AIFM2, BBC3, BCL2L1, GADD45A, PMAIP1, TNFRSF10B*; cell cycle genes: *CDKN1A*; inhibition of angiogenesis and metastasis genes: *CD82, SERPINE1*), while decreased the expression of p53-repressed genes (such as cell cycle genes: *CCNB1, CCNB2, CCND3, CDK1, CDK2, GTSE1*; DNA damage and repair prevention gene: *RRM2*) (Fig. 3E) [25–29]. Western Blotting of typical p53 signaling pathway proteins also verified that the knockdown of TACC3 up-regulated the expression of CDKN1A (p21) and cleaved caspase 3, and down-regulated the expression of RRM2 [30–32] (Fig. 3F). These data support that TACC3 knockdown activates the p53 signaling pathway in PDAC cells, which also suggests the pro-tumorigenesis role of TACC3.

To further explore the role of TACC3 in the spindle assembly in PDAC cells, we performed IF staining for α-tubulin in Panc-1 cells transfected with scrambled shRNA and TACC3 shRNA to examine spindle morphology [33, 34]. The results showed that while most scrambled shRNA-transfected Panc-1 cells showed typical bipolar spindle assembly, abnormal spindle assembly (distorted, multipolar, or monopolar spindles) was frequently observed in TACC3-deficient Panc-1 cells (Fig. 3G, H). This was consistent with our hypothesis that TACC3 protein deficiency compromises spindle assembly in Panc-1 cells. IF staining of previously obtained frozen sections of subcutaneous tumor tissue also showed that knockdown of the TACC3 protein under in vivo conditions interfered with normal spindle assembly in Panc-1 cells, with a marked increase in cells with abnormal spindle morphology (Fig. 3I, J). These results suggest that TACC3 is essential for normal bipolar spindle formation in Panc-1 cells, while TACC3 deficiency leads to abnormal spindle assembly, hindering the division and proliferation of Panc-1 cells. Overall, TACC3 is critical for the correct assembly of bipolar spindles in PDAC cells. Mis-assembly of the spindle due to TACC3 deficiency hinders progression of the cell cycle and limits cell division and proliferation.

### TACC3 physically interacts with and stabilizes KIF11 in Panc-1 cells

**Fig. 4:**
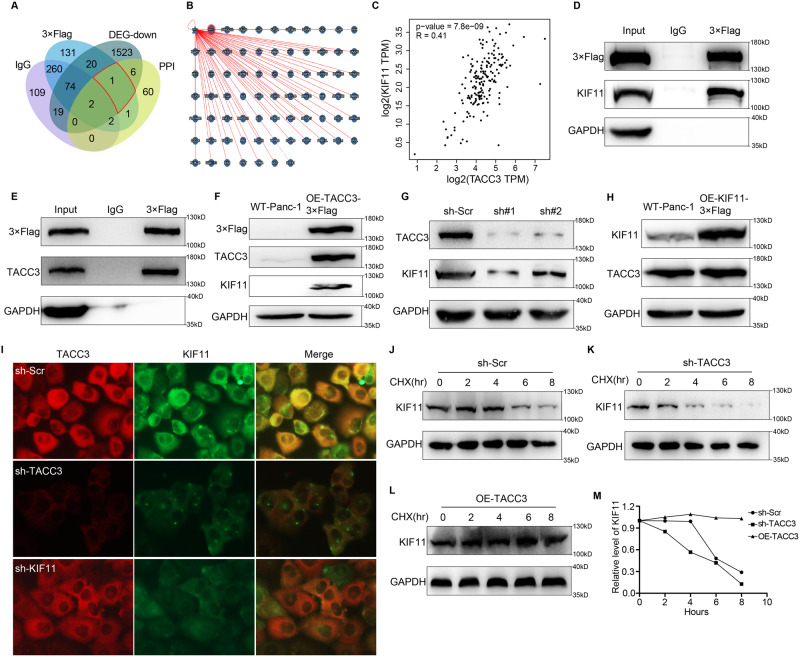
TACC3 physically interacts with and stabilizes KIF11 in Panc-1 cells. HEK293T cells were infected with 3×Flag-TACC3 plasmids and whole cell lysate was collected, an IgG antibody specific to 3×Flag and a normal IgG were respectively added to form co-immunoprecipitation, and the types of proteins in the precipitation were identified by mass spectrometry (MS). Proteins identified in 3×Flag-precipitates but not in normal IgG-precipitates are proteins that interact with TACC3. **A** Venn diagram, including 4 parts of data: (1) In the above Co-IP experiment, the proteins interacting with normal IgG (in purple color); (2) In the above Co-IP experiment, proteins interacting with 3×Flag-TACC3 (in blue color); (3) Proteins interacting with TACC3 in the PPI Network database (in yellow color); (4) down-regulated genes caused by TACC3 deficiency obtained by RNA sequencing analysis (in gray color). The above data were comprehensively analyzed and the downstream protein KIF11 (in red wire frame) which may interact with TACC3 was obtained. **B** Proteins that interact with TACC3 in PPI Network online database. **C** The correlation between TACC3 and KIF11 in PDAC obtained from GEPIA online database (Pearson correlation coefficient, *R* = 0.41, *p*-value = 7.8e −09). **D** The 3×Flag-TACC3 plasmid was transfected into HEK293T cells, and whole cell lysate was collected. Specific anti-3×Flag IgG antibody and normal IgG were respectively added to form co-immunoprecipitation. The expression levels of 3×Flag-TACC3 and KIF11 in whole cell lysis products (input), immune-precipitates produced by 3×Flag specific antibody (3×Flag), and immune-precipitates produced by normal IgG (IgG) were examined by Western blotting. **E** The 3×Flag-KIF11 plasmid was transfected into HEK293T cells, and whole cell lysate was collected. Specific anti-3×Flag IgG antibody and normal IgG were respectively added to form co-immunoprecipitation. The expression levels of TACC3 and 3×Flag-KIF11 in whole cell lysis products (input), immune-precipitates produced by 3×Flag specific antibody (3×Flag), and immune-precipitates produced by normal IgG (IgG) were examined by Western blotting. **F** 3×Flag-TACC3 was overexpressed in Panc-1 cells through lentiviral infection. Western blotting showed that TACC3 overexpression was successfully constructed, the KIF11 expression was uniformly increased, and the change of 3×Flag expression level was consistent with that of TACC3. **G** Panc-1 cells were transfected with TACC3 shRNA. Western blotting showed that TACC3 expression was decreased and the KIF11 expression was decreased uniformly. **H** 3×Flag-KIF11 was overexpressed in Panc-1 cells through lentiviral infection. Western blotting showed that KIF11 overexpression was successfully constructed. However, TACC3 expression did not change with that of KIF11. **I** IF staining of TACC3 (in red color) and KIF11 (in green color) proteins on control Panc-1 cells (sh-Scr), TACC3-deficient Panc-1 cells (sh-TACC3) and KIF11-deficient Panc-1 cells (sh-KIF11). **J**–**M** Control Panc-1 cells (**J**), TACC3-deficient Panc-1 cells (**K**) and TACC3-overexpressed Panc-1 cells (**L**) were treated with CHX for 8 h and collected every 2 h to examine the KIF11 levels by western blotting. GAPDH was used as a loading control. (**M**) Line chart presented the change of KIF11 protein level over time under CHX treatment. (Values are shown as the means ± SDs. *n* = 3. All studies were performed with three biological replicates).

Next, we performed Co-IP coupled with mass spectrometry (MS)-dependent protein identification to further explore the mechanisms by which the TACC3 protein regulates spindle formation. We generated a Venn diagram by using the following data: (1) proteins that interacted with TACC3 in a Co-IP experiment (Supplementary Table 3), (2) proteins that interacted with TACC3 in the PPI Network online database (Fig. 4B), and (3) genes that were downregulated by TACC3 deficiency in RNA sequencing analysis; we ultimately identified the protein Kinesin Family Member 11 (KIF11, also known as Eg5) as an interaction partner of TACC3 (Fig. 4A). KIF11 is a microtubule plus end-directed motor protein that functions in mitosis to cross-link antiparallel microtubules and slide them apart [35, 36]. KIF11 establishes and maintains spindle bipolarity by generating outward forces and contributes to microtubule flux, which plays a crucial role in the assembly and function of the spindle. Data from the GEPIA online database suggested that the expression of KIF11 is positively correlated with TACC3 in PDAC tissues (Fig. 4C). The above-described data mining results suggest that TACC3 physically interacts with and regulates KIF11.

To verify the existence of physical interactions between TACC3 and KIF11, we conducted a Co-IP of TACC3 and KIF11. As shown in Fig. 4D, KIF11 was readily precipitated in 3×Flag-TACC3-infected HEK293T cells using an IgG antibody against 3×Flag. Similarly, TACC3 was also readily precipitated in 3×Flag-KIF11-infected HEK293T cells using an IgG antibody against 3×Flag (Fig. 4E). However, normal IgG failed to precipitate TACC3 or KIF11. These results indicate that TACC3 physically interacts with KIF11.

Next, we examined whether the expression of the KIF11 protein is regulated by TACC3. First, we overexpressed 3×Flag-TACC3 in Panc-1 cells via lentiviral infection and measured the expression level of KIF11 by Western blotting. As shown in Fig. 4F, the overexpression of TACC3 was accompanied by elevated expression of KIF11. Moreover, the change in the 3×Flag expression level was consistent with that of TACC3, suggesting that the expression level of TACC3 could be reflected by 3×Flag. After the transfection of TACC3 shRNA into Panc-1 cells, KIF11 expression was also consistently decreased (Fig. 4G). These results suggested that TACC3 can regulate the expression of KIF11. However, the overexpression of KIF11 in Panc-1 cells did not cause increased expression of TACC3 (Fig. 4H). In addition, IF staining of TACC3 (in red color) and KIF11 (in green color) proteins was performed on the control cells (sh-Scr), TACC3-deficient cells (sh-TACC3) and KIF11-deficient cells (sh-KIF11) to further verify the interaction between TACC3 and KIF11. As shown in Fig. 4I, TACC3 and KIF11 showed an obvious colocalization. Compared with control cells, the fluorescence intensity of KIF11 protein was significantly reduced in TACC3-deficient cells, suggesting an adjoint down-regulation of KIF11 expression after the knockdown of TACC3. However, the fluorescence intensity of TACC3 in KIF11-deficient cells did not decrease, suggesting that the knockdown of KIF11 did not in turn lead to the down-regulation of TACC3 expression. Western Blotting and IF staining showed the consistent results. These data suggest that KIF11 is a downstream protein of TACC3 and that its expression level is regulated by TACC3.

During meiosis in mammalian oocytes, TACC3 plays an important role in the composition of the spindle, and TACC3 depletion results in severe exhaustion of both the microtubules bound to chromosomal kinetochores and the microtubules overlapping in an antiparallel manner in the spindle midzone [8]. Therefore, we speculated that TACC3 may also play a role in stabilizing KIF11 in Panc-1 cells. To further verify the regulatory effect of TACC3 on the stability of the KIF11 protein, we performed a time-course experiment to monitor KIF11 degradation in TACC3-deficient Panc-1 cells (sh-TACC3), TACC3-overexpressing Panc-1 cells (OE-TACC3) and control Panc-1 cells (sh-Scr) under cycloheximide (CHX) treatment [37, 38]. As shown in Fig. 4J, the level of KIF11 showed a gradual decline over 4–6 h following CHX treatment in control Panc-1 cells. However, in TACC3-deficient Panc-1 cells, the level of KIF11 dramatically declined within 2 h following CHX treatment (Fig. 4K). In TACC3-overexpressing Panc-1 cells, the level of KIF11 remained unchanged for 8 h in the presence of CHX (Fig. 4L). The line chart presents the change in KIF11 protein levels over time under CHX treatment (Fig. 4M). These results proved the role of TACC3 in stabilizing KIF11. Taken together, the results show that KIF11 is a downstream protein of TACC3 and that TACC3 physically interacts with and stabilizes KIF11.

### KIF11 is essential for proliferation and spindle formation in PDAC cells

**Fig. 5:**
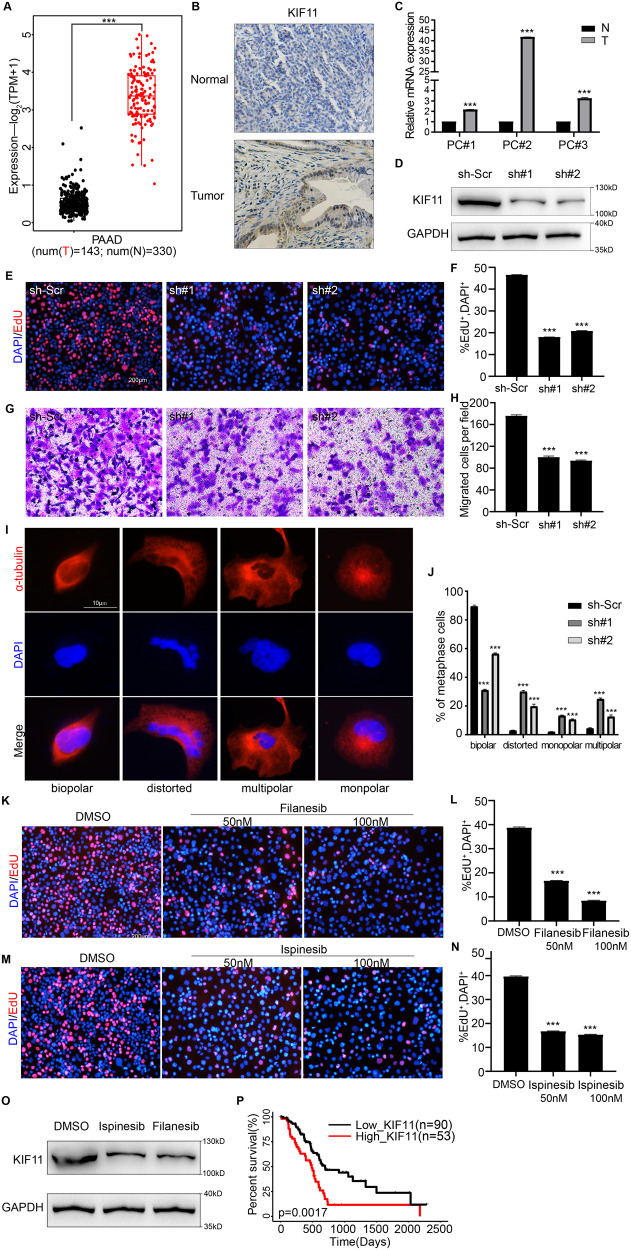
KIF11 is essential for proliferation and spindle formation of PDAC cells. **A** Differential expression of KIF11 between different disease states (Tumor or Normal) in PDAC was analyzed by TCGA and GTEx database. **B** Representative images of negative KIF11 IHC staining in normal pancreas and representative images of strong positive KIF11 staining in PDAC tissues. Scale bar: 100 µm. **C** qPCR analysis of KIF11 mRNA expression levels in three paired PDAC tissues (T) and adjacent normal pancreatic tissue (N). **D**–**J** Panc-1 cells were infected with scrambled shRNA (sh-Scr) or KIF11 shRNA (sh#1, sh#2). **D** Western blotting detected the KIF11 protein levels in Panc-1 cells to verify transfection efficiency. **E** Representative images of EdU assays detecting the proliferation of control Panc-1 cells and KIF11-deficient cells, and (**F**) the percentage of EdU^+^ cells (in red color) per field was quantified. Scale bar: 200 µm. **G** Representative images of Transwell assays detecting the migration of control cells and KIF11-deficient cells, and (**H**) the number of migrated cells per field was counted. Scale bar: 200 µm. **I** Spindle morphology of Panc-1 cells at metaphase of mitosis were detected by IF staining with an anti- α-tubulin antibody (in red color). Nuclei were counterstained with DAPI (in blue color). Scale bar: 10 µm. **J** The percentage of Panc-1 cells with normal (bipolar) or abnormal spindles (including distorted, monopolar, or multipolar) was quantified. **K**–**L** Panc-1 cell lines were treated with DMSO, 50 nM filanesib, or 100 nM filanesib for 48 h under in vitro conditions. **K** Representative images of EdU assays detecting the proliferation of the three groups and (**L**) the percentage of EdU^+^ cells (in red color) per field was quantified. **M**–**N** Panc-1 cell lines were treated with DMSO, 50 nM ispinesib, or 100 nM ispinesib for 48 h under in vitro conditions. **M** Representative images of EdU assays detecting the proliferation of the three groups and (**N**) the percentage of EdU^+^ cells (in red color) per field was quantified. **O** Panc-1 cells treated with DMSO, 100 nM ispinesib, or 100 nM filanesib for 48 h were collected and Western blotting was used to examined for KIF11 expression; using GAPDH as a loading control. **P** TCGA database analysis showed that high KIF11 expression is associated with poor prognosis in PDAC (log-rank test, *P* = 0.0017). (Values are shown as the means ± SDs. *P < 0.05, **P < 0.01, ***P < 0.001, *n* = 3 per group. All studies were performed with three biological replicates).

Next, we explored the specific role of KIF11 in the malignant phenotype of PDAC. First, we found that KIF11 expression was significantly up-regulated in PDAC tissue samples compared to normal pancreatic tissue samples using TCGA and GTEx database (Fig. 5A). Next, we used IHC staining to evaluate KIF11 abundance in 46 pairs of PDAC tissues and adjacent normal pancreatic tissues from our center. IHC results showed that the KIF11 protein was barely detectable in normal pancreatic tissues, but was strongly expressed in PDAC tissues (Fig. 5B). At the same time, paired PDAC tissues and normal pancreatic tissues from 3 patients were collected and qPCR detection showed that KIF11 expression in cancer tissues was significantly higher than that in normal tissues (Fig. 5C). These results indicate that KIF11 is clearly differentially expressed in PDAC and normal pancreas.

Next, we investigated changes in cellular function after knocking down KIF11 expression in Panc-1 cells. We infected Panc-1 cells with a lentivirus expressing KIF11-specific shRNA (sh#1 and sh#2), and a scrambled shRNA (sh-Scr) was used as a control. Western blotting was used to verify the knockdown efficiency (Fig. 5D). Next, we compared the proliferation ability of control cells (sh-Scr) and KIF11-deficient cells (sh#1 and sh#2) using EdU assays. The results showed that KIF11 knockdown significantly inhibited the growth of Panc-1 cells in vitro (Fig. 5E, F). In addition, Transwell assays showed that KIF11 knockdown significantly inhibited the migration ability of Panc-1 cells in vitro (Fig. 5G, H). These results suggest that KIF11 promotes the malignant phenotype of PDAC cells.

To explore whether KIF11 is involved in regulating mitosis and spindle formation in PDAC cells, IF staining for α-tubulin was performed in Panc-1 cells at metaphase during mitosis to examine the spindle morphology. As shown in Fig. 5I, J, most of the cells in the control group showed a typical bipolar spindle assembly, while most of the cells with KIF11 deficiency showed an abnormal spindle assembly (distorted, multipolar, or monopolar spindle). These findings suggest that KIF11 is essential for bipolar spindle formation in PDAC cells, which is consistent with our previous knowledge of the role of KIF11 in establishing and maintaining the spindle bipolarity.

Filanesib and ispinesib are effective inhibitors of KIF11 and have been shown to have antitumor effects in meningiomas [39, 40]. Panc-1 cells were treated with DMSO, 50 nM filanesib, or 100 nM filanesib for 48 h. Cell proliferation was detected by EdU assays. As shown in Fig. 5K, L, the proliferation capacity of Panc-1 cells decreased significantly after filanesib treatment in a concentration-dependent manner. The same phenomenon also occurred after ispinesib treatment, as shown in Fig. 5M, N. Subsequently, we collected Pan-1 cells treated with DMSO, 100 nM ispinesib, or 100 nM filanesib for 48 h and detected the expression of KIF11 by Western blotting (Fig. 5O). We found that ispinesib and filanesib significantly down-regulated KIF11 expression. This finding suggests that down-regulation of KIF11 expression by inhibitors weakens the proliferation ability of PDAC cells, which may provide hope and new approaches for the treatment of PDAC. Finally, through downloading and analyzing the 143 clinical samples of PDAC from the TCGA database, we found that high expression of KIF11 was significantly associated with poor prognosis of PDAC (Fig. 5P), which is consistent with our experimental results. Collectively, the above-mentioned data demonstrate the essential role of KIF11 in proliferation and spindle formation in PDAC cells and show that the pharmacological inhibition of KIF11 can suppress the above-mentioned effects.

### TACC3 inhibitors suppress the progression of pancreatic cancer in vivo

**Fig. 6:**
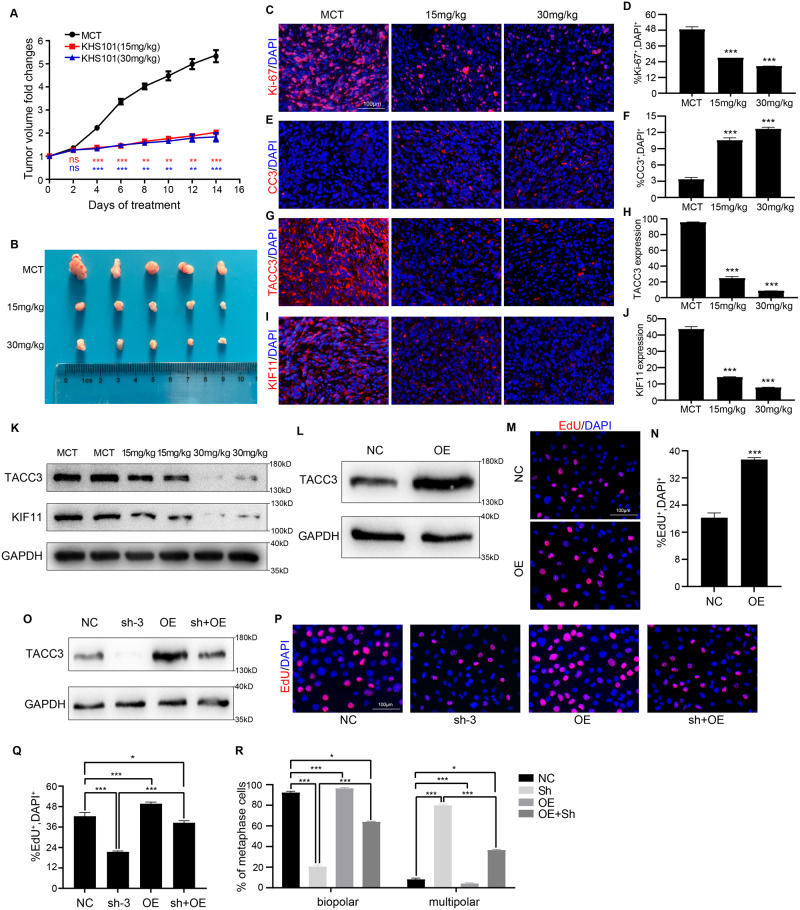
TACC3 inhibitors suppress the progression of PDAC in vivo. **A**–**K** To explore whether decreasing TACC3 protein expression affected tumor progression in PDAC, we constructed a mouse subcutaneous tumor model of Panc-1 cells. When the tumor volume reached ~200 mm^3^, the mice were randomly grouped and daily intraperitoneal administration was performed by a concentration gradient of MCT, 15 mg/kg KHS101, or 30 mg/kg KHS101. *n* = 5 biologically independent mice per group. **A** Volume changes of subcutaneous tumor was monitored every 2 days and displayed by line charts. **B** On the 14th day of MCT/KHS101 treatment, tumors were separated and photographed to show tumor size. Frozen sections were made from subcutaneous tumor tissue. IF staining with antibodies to Ki-67 (in red color, (**C**)) and CC3 (in red color, (**E**)) was performed to detect proliferation and apoptosis. Nuclei were counterstained with DAPI (in blue color) and the percentage of Ki-67^+^ (**D**) or CC3^+^ cells (**F**) per field was quantified. IF staining with antibodies to TACC3 (in red color, (**G**)) and KIF11 (in red color, (**I)**) was performed to evaluate protein expression. Nuclei were counterstained with DAPI (in blue color) and protein abundance of TACC3 (**H**) and KIF11 (**J**) was summarized. Scale bar: 100 µm. **K** Subcutaneous tumor tissue was collected from the three groups, and TACC3 or KIF11 expression was detected by Western blotting; GAPDH was used as a loading control. **L**–**N** TACC3 was overexpressed in H6C7 human normal pancreatic duct epithelial cells. **L** TACC3 overexpression (OE) was verified by Western blotting; normal H6C7 cells were used as a negative control (NC), and GAPDH was used as a loading control. **M** EdU assays was performed to detect the proliferation of normal H6C7 cells and TACC3-overexpressed H6C7 cells, and (**N**) the percentage of EdU^+^ cells (in red color) per field was quantified. Scale bar: 100 µm. **O**–**R** Wild type Panc-1 cells (NC) were used as a control group, and TACC3-overexpression Panc-1 cells (OE) were constructed. By using a TACC3 shRNA targeting the TACC3 non-coding region 3′ UTR, a TACC3-knockdown Panc-1 cells (sh-3) was constructed. In sh-3, TACC3 was overexpressed to rescue the TACC3 expression level (sh+OE). **O** TACC3 expression in the four groups of cells was verified by Western blotting. GAPDH was used as a loading control. **P** EdU assays were performed to detect the proliferation of the four groups of cells, and (**Q**) the percentage of EdU^+^ cells (in red color) per field was quantified. Scale bar: 100 µm. **R** Spindle morphology at metaphase of mitosis was detected by IF staining using an anti-α-tubulin antibody. Nuclei were counterstained with DAPI. The percentage of the four groups of cells with bipolar or multipolar spindles was quantified. (Values are shown as the means ± SDs. *P < 0.05, **P < 0.01, ***P < 0.001. Unless otherwise specified, *n* = 3. All studies were performed with three biological replicates).

KHS101 is an effective inhibitor of TACC3 [41–44]. Treatment of rat hippocampal neural progenitor cells with KHS101 can significantly reduce the TACC3 level and inhibit the progression of nasopharyngeal carcinoma [41]. In glioblastoma, a series of analogs of KHS101 have been shown to have significant anti-tumor cell proliferation activity by inhibiting TACC3 functions and are expected to be developed for the treatment of glioblastoma [42]. To investigate whether reducing TACC3 protein expression in PDAC can inhibit tumor progression, we constructed a mouse subcutaneous tumor model with Panc-1 cells and treated the mice daily with a concentration gradient of MCT, 15 mg/kg KHS101, or 30 mg/kg KHS101. By recording tumor volume changes, we found that KHS101 significantly inhibited the progression of PDAC in mice (Fig. 6A, B). All tumor specimens were collected after 14 days of treatment. Frozen sections were generated from these subcutaneous tumor tissues, and IF staining of Ki-67 and CC3 was performed to detect the proliferation and apoptosis of PDAC cells. We found that compared with those in the control mice (MCT-treated), PDAC cells in KHS101-treated mice showed significantly reduced proliferation (Fig. 6C, D) and enhanced apoptosis (Fig. 6E, F). Furthermore, through IF staining of TACC3 and KIF11 in subcutaneous tumors, we verified that KHS101 treatment significantly reduced the TACC3 expression (Fig. 6G, H), and this decrease was accompanied by a decrease in KIF11 expression (Fig. 6I, J). These results indicate that the application of a TACC3 inhibitor in mice to reduce the expression level of the TACC3 protein can effectively inhibit the progression of PDAC, which is expected to provide new ideas for the treatment of PDAC. The same trend of TACC3 or KIF11 expression level was verified by the Western blotting of collected subcutaneous tumor tissue (Fig. 6K). The above results suggested that KHS101 can significantly reduce the TACC3 expression in PDAC and inhibit tumor progression.

Next, we conducted TACC3 rescue experiments. First, we overexpressed TACC3 in H6C7 normal human pancreatic duct epithelial cells to observe the effect on proliferation. The efficiency of TACC3 overexpression was verified by Western blotting (Fig. 6L). EdU assays showed that TACC3 overexpression resulted in increased cell proliferation (Fig. 6M, N). Therefore, next, we overexpress TACC3 in TACC3-deficient cells to see whether it can rescue the proliferation restriction caused by TACC3 knockdown. Wild-type Panc-1 cells (NC) were used as a control group, and TACC3-overexpressing Panc-1 cells (OE) were constructed. Next, by using a TACC3 shRNA which targets the TACC3 non-coding region 3′ UTR, we constructed TACC3-knockdown Panc-1 cells (sh-3). In sh-3 group, TACC3 was overexpressed to rescue the TACC3 expression level (sh+OE). By using Western blotting, the successful construction of three groups of stable strains (sh-3, OE, sh+OE) was verified (Fig. 6O). The EdU assays results showed that rescuing the TACC3 expression in TACC3-knockdown cells rescues the proliferation ability of Panc-1 cells (Fig. 6P, Q). Furthermore, IF staining and statistical analysis of spindle morphology at metaphase during mitosis also showed that rescuing TACC3 expression rescued correct spindle assembly in Panc-1 cells (Fig. 6R). The above experiments indicate that the proliferation and progression of PDAC cells can be inhibited by reducing TACC3 expression in vivo and in vitro, while increasing TACC3 expression results in enhanced cell proliferation. In conclusion, TACC3 is crucial for the proliferation of PDAC cells and for the development of PDAC both in vitro and in vivo.

### TACC3 deficiency increases the sensitivity of PDAC cells to chemotherapy drugs

**Fig. 7:**
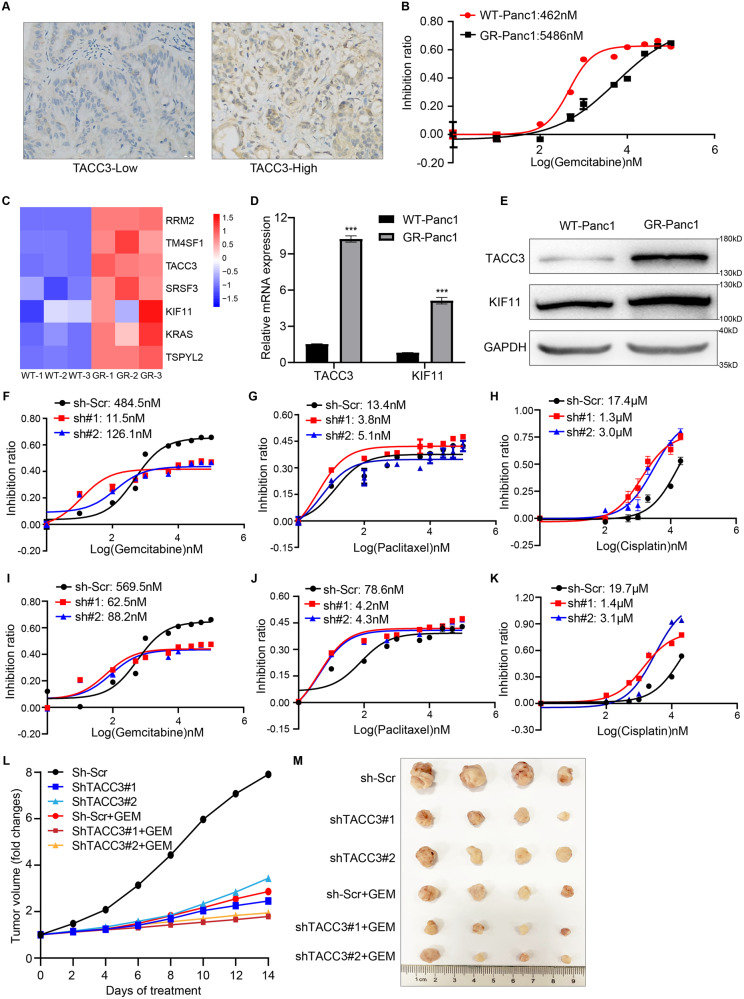
TACC3 deficiency increases the sensitivity of PDAC cells to chemotherapy drugs. **A** Representative images of high/low TACC3 protein expression in PDAC samples from patients with neoadjuvant therapy. Scale bar: 100 µm. **B**–**E** Panc-1 cells was cultured with repeated gemcitabine induction to generate gemcitabine-resistant (GR) sublines. **B** The successful construction of gemcitabine-resistant Panc-1 sublines was verified by examining IC50 in parental cells versus GR sublines. Gene expression in gemcitabine-sensitive cell lines (WT) and GR sublines was analyzed by RNA sequencing. **C** Cluster heatmaps showed the relative expression levels of TACC3, KIF11 and some verified gemcitabine-resistance related genes in WT and GR sublines. **D** qPCR verified the differential expression of TACC3 and KIF11 in WT and GR sublines. **E** Western blotting validated the differential expression of TACC3 and KIF11 in WT and GR sublines. **F**–**H** Panc-1 cells were infected with scrambled shRNA (sh-Scr) or TACC3 shRNA (sh#1, sh#2) to construct stable strains. IC50 of gemcitabine (**F**) paclitaxel (**G**) and cisplatin (**H**) in these three group of cells was quantified. **I**–**K** Panc-1 cells were infected with scrambled shRNA (sh-Scr) or KIF11 shRNA (sh#1, sh#2) to construct stable strain. IC50 of gemcitabine (**I**) paclitaxel (**J**) and cisplatin (**K**) in these three group of cells was quantified. **L**–**M** To explore the combination effect of TACC3 knockdown plus chemotherapy in vivo, we constructed a mouse subcutaneous tumor model of Panc-1 cells. *n* = 4 biologically independent mice per group. Each group of mice were fed with PBS containing 2 μg/mL DOX to regulate the TACC3 expression (sh-Scr, shTACC3#1, shTACC3#2). Meanwhile, the combination treatment group (TACC3 + GEM) was intraperitoneally injected with 20 mg/kg gemcitabine in a PBS vehicle, and the non-combination treatment group was treated with PBS only. **L** Volume changes of subcutaneous tumor was monitored every 2 days and displayed by line charts. **M** On the 14th day of treatment, tumors were separated and photographed to show tumor size. (Unless otherwise specified, *n* = 3. All studies were performed with three biological replicates).

Chemotherapy is an important adjuvant treatment for all stages of PDAC, but the prevalence of drug resistance often leads to poor therapeutic outcomes. RRM2 is an important gemcitabine resistance-related gene in PDAC [32, 45]. In previous studies, we found that knockdown of TACC3 led to the down-regulation of RRM2. Therefore, we wonder whether TACC3 may be associated with chemotherapy resistance in PDAC. At present, tumor response to preoperative therapies is mainly measured by radiographic metrics and serum cancer antigen (CA) 19-9 level [46, 47]. The Response Evaluation Criteria in Solid Tumors (RECIST, version 1.1) proposes the radiographic guideline for evaluating tumor response to chemotherapy, and the changes are divided into four grades: complete response (CR), partial response (PR), progressive disease (PD) and stable disease (SD) [48, 49]. We performed IHC staining of TACC3 in tumor tissues from 54 patients who received neoadjuvant therapy at our center. Patients were divided into a high-TACC3 expression cohort (*n* = 28) and a low-TACC3 expression cohort (*n* = 26) based on TACC3 expression (Fig. 7A). We found that higher TACC3 expression was associated with lower radiographic and serological response rates in PDAC patients with chemotherapy (Table 2, Supplementary Table 4). These findings prompted us to further investigate the role of TACC3 in chemotherapy resistance of PDAC. Gemcitabine, paclitaxel and cisplatin are all first-line drugs for PDAC chemotherapy; thus, we wanted to further clarify the relationship between TACC3 expression and sensitivity to these typical chemotherapy drugs.

**Table 2:** Metrics of response to chemotherapy among 54 patients receiving neoadjuvant chemotherapy.

Characteristics	The Pooled (*N* = 54)	TACC3		*P*-value
		Low (*N* = 26)	High (*N* = 28)	
Radiographic measures after treatment				
Reduction in primary tumor volume				0.041
No	20	6	14	
Yes	34	20	14	
RECIST 1.1				0.041
CR	0	0	0	
PR	29	18	11	
SD	24	8	16	
PD	1	0	1	
Serologic measures after treatment				
Change in CA 19-9				0.025
Normal to normal	8	6	2	
Elevated to normal	17	11	6	
Elevated to elevated	29	9	20	
Normal to elevated	0	0	0	

First, we cultured Panc-1 cells with repeated gemcitabine induction to generate gemcitabine-resistant sublines. As shown in Fig. 7B, the half-maximum inhibitory concentration (IC50) value of gemcitabine in the resistant subline was significantly higher than that in the parental cells. This result proved that we successfully constructed gemcitabine-resistant Panc-1 strains. Next, we collected these cells and performed RNA sequencing to analyze the gene expression difference between gemcitabine-resistant and gemcitabine-sensitive cell lines (Supplementary Table 5). Previous studies reported that high expression of RRM2, TM4SF1, SRSF3, and TSPYL2 promoted drug resistance in PDAC, and in our results, these genes were also consistently highly expressed in our gemcitabine-resistant sublines [50–52] (Fig. 7C). Moreover, we also found the high expression of TACC3 and KIF11, which have not been reported yet. Meanwhile, by qPCR and Western blotting, we verified that the expression of TACC3 and KIF11 was significantly increased in gemcitabine-resistant cell sublines (Fig. 7D, E). These data indicated a correlation between high expression of TACC3 and KIF11 and gemcitabine resistance in PDAC.

Next, we measured the IC50 of gemcitabine/paclitaxel/cisplatin in Panc-1 cells transfected with scrambled shRNA (sh-Scr) and TACC3 shRNA (sh#1 and sh#2). The results showed that TACC3-deficient Panc-1 cells had significantly increased sensitivity to all three drugs (Fig. 7F–H). Similarly, compared to that in scrambled shRNA-transfected Panc-1 cells (sh-Scr), the IC50 of gemcitabine/paclitaxel/cisplatin was also significantly decreased in Panc-1 cells transfected with KIF11 shRNA (sh#1 and sh#2) (Fig. 7I–K), indicating an increased sensitivity to these three drugs. In addition, we constructed a subcutaneous tumor model to explore the combination effect of TACC3 knockdown plus gemcitabine chemotherapy. BALB/c-nu mice were randomly divided into 6 groups, and subcutaneous tumors were established with Panc-1 cells. The expression of TACC3 was regulated by the DOX-inducible Tet-On system as described previously. Each group of mice were fed with PBS containing 2 μg/mL DOX. Meanwhile, the combination treatment group was intraperitoneally injected with 20 mg/kg gemcitabine in a PBS vehicle, while the non-combination treatment group was treated with PBS only. Tumor size was measured and recorded every 2 days. All tumor specimens were collected after 14 days of treatment. We found that, consistent with previous results, TACC3 deficiency significantly suppressed the tumor growth in vivo. In addition, the combination of TACC3 knockdown plus gemcitabine chemotherapy further inhibited the tumor progression (Fig. 7L, M). These are very encouraging findings, suggesting that that knockdown of TACC3/KIF11 can promote the sensitivity of PDAC cells to classical chemotherapy drugs.

## Discussion

PDAC is one of the most dangerous cancers threatening human health, and its incidence is increasing annually, with an estimated total of 62,210 new cases in 2022 [2]. The progression of PDAC is rapid and aggressive, with 80% to 85% of patients having already developed distant metastases at the initial visit [1]. However, treatment options are quite limited. At present, surgical resection is the only radical cure, but there is a high recurrence rate. Postoperative adjuvant chemotherapy has become a routine treatment. Nevertheless, there has been no promising progress toward a cure for PDAC, and PDAC has an overall 5-year survival rate of only 10%. In-depth exploration of the biological mechanisms underlying the pathogenesis and progression of PDAC is expected to identify potential targets for disease treatment, which could solve this important scientific problem.

Our current study explores the specific role of TACC3 in PDAC. By performing database mining and statistical analysis of tissue samples, we demonstrated that TACC3 was highly expressed in PDAC and was associated with poor prognosis. Knockdown of TACC3 by shRNA induced S phase arrest in PDAC cells, which resulted in decreased cell proliferation and migration and increased cell apoptosis both in vitro and in vivo. By performing RNA sequencing of Panc-1 cells transfected with scrambled shRNA and TACC3 shRNA and subsequent GO and KEGG enrichment analysis of the differentially expressed genes, we found that the regulatory effect of TACC3 on the cell cycle was achieved via effects on spindle assembly. Consistent with our findings, a recent study demonstrated that TACC3 is an essential component of the human oocyte microtubule organizing center whose disruption results in spindle assembly defects and oocyte maturation arrest. It’s worth mentioning that Huo et al. have observed an opposite phenomenon as the inhibition of TACC3 promoted the proliferation and migration of cancer cells in vitro in breast cancer [53]. We deduce several possible reasons. First, in different tumor types, or at different stages of tumor development, cancer cells may exhibit different biological characteristics, or the tumor microenvironment may exert its impact. For example, in prostate cancer, osteosarcoma and PDAC [54, 55], inhibition of TACC3 restrains growth, proliferation or migration of cancer cells, while in breast cancer it leads to an opposite effect as is observed by Huo et al. Secondly, in various tumor types, the dominant molecular mechanism of TACC3 may be different, which may lead to opposite phenotypes. Our study found that TACC3 regulates the progression of PDAC primarily through its involvement in spindle assembly. While the role of TACC3 in breast cancer revealed by Huo et al. mainly involves Notch4 and CDH5-related signaling pathways, as well as the Wnt/β-catenin and Notch signaling pathways. Interestingly, even in breast cancer, the study by Saatci et al. showed that TACC3 knockdown inhibited the proliferation of cancer cells by affecting spindle assembly and mitosis.

Our study also identified the downstream protein interacting with TACC3 as KIF11. TACC3 regulates and stabilizes the expression level of KIF11. Previous studies have demonstrated that KIF11 is a microtubule plus end-directed motor protein that functions in mitosis by cross-linking antiparallel microtubules and sliding them apart [35, 36]. KIF11 establishes and maintains the spindle bipolarity by generating outward forces and contributes to microtubule flux, which plays a crucial role in the assembly and function of the spindle [56, 57]. Our study found the essential functions of KIF11 in spindle formation and cell proliferation in PDAC cells and showed that its expression is regulated and stabilized by TACC3. When TACC3 is knocked down, the expression of KIF11 is decreased accordingly. A similar phenomenon occurs during meiosis in mammalian oocytes, in which TACC3 depletion results in severe exhaustion of both the microtubules bound to chromosomal kinetochores and the microtubules overlapping in an antiparallel manner in the spindle midzone [8]. To further clarify the physical binding sites of the TACC3 protein and KIF11 protein, we constructed CDS region truncates of these two genes respectively based on the UniProt website. Unfortunately, due to the non-expression of truncations, our study did not identify the specific sites where TACC3 interacts with KIF11, which still needs to be further explored.

Moreover, we found that KHS101, an inhibitor of TACC3, showed promising results in the treatment of PDAC. The application of KHS101 in mice significantly inhibited the progression of subcutaneously transplanted tumors and showed dose-dependent characteristics. Previously, KHS101 was shown to play a role in tumor therapy by down-regulating TACC3 levels in breast cancer, nasopharyngeal carcinoma and meningioma [41–43]. Other compounds based on KHS101 have also been developed and have shown even more potent anti-cancer effects. These data suggest that TACC3 may become an important therapeutic target for PDAC, which is expected to provide hope and a new direction for PDAC treatment. Finally, the knockdown of TACC3 enhanced the sensitivity of PDAC to chemotherapy drugs both in vitro and in vivo, which suggested that an effective combination of chemotherapeutic drugs and targeted therapy for TACC3 may achieve better efficacy than monotherapy.

In our study, KIF11 and TACC3 were also shown to be implicated in PDAC cell migration. This may be related to the properties of TACC3/KIF11 as motor proteins, which are closely related to the cell mobility and migration [58, 59]. TACC3 regulates and stabilizes the microtubule of the cytoskeleton. From our previous studies, we can see that the of knockdown TACC3 led to the down-regulated expression of a variety of microtubule, including KIF15, KIF20A, KIF11 and so on, which closely take part in the cell migration [60–62] (Supplementary Table 2). Our study demonstrates that TACC3 regulates and stabilizes the expression of KIF11. In previous studies, KIF11 has been found to enhance cell migration of fibroblasts by triggering the myosin II-associated signaling pathway [63]. Therefore, further exploration of this pathway in the future may help to well explain our migration model in PDAC cells.

**Fig. 8:**
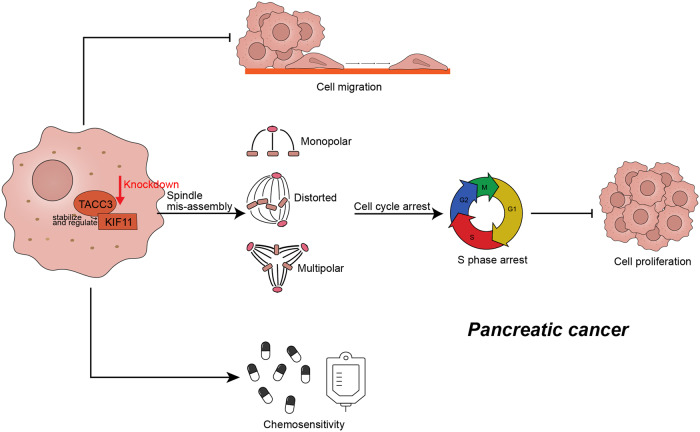
Graphical diagram of TACC3 knockdown inhibits tumor cell proliferation and migration, and increases chemosensitivity in pancreatic cancer. TACC3 is a promoter of PDAC that promotes the cancer cell proliferation by regulating bipolar spindle formation and cell cycle progression, and promotes cell migration and chemoresistance as well. TACC3 physically interacts with, stabilizes and regulates its downstream protein KIF11, which establishes and maintains the spindle bipolarity by generating outward forces and contributes to microtubule flux. Knockdown of TACC3 and its downstream protein KIF11 leads to abnormal spindle formation and S phase arrest of the cell cycle, inhibiting cancer cell proliferation, and suppresses cancer cell migration and increases chemosensitivity in PDAC.

In summary, our study demonstrated that TACC3 is a promoter of PDAC that promotes progression of the cell cycle and supports the malignant phenotype of PDAC cells by regulating bipolar spindle formation. Knockdown of TACC3 and its downstream protein KIF11 inhibits tumor cell proliferation and increases chemosensitivity in PDAC (Fig. 8). Our findings suggest that TACC3 could serve as a novel molecular marker and a promising therapeutic target for PDAC.

### Supplementary information

Supplementary Material Legends

Supplementary Table 1

Supplementary Table 2

Supplementary Table 3

Supplementary Table 4

Supplementary Table 5

original data files

Reproducibility checklist

## Data Availability

Supplementary Table 1 provides the clinical data of 218 patients included in the TMA. Supplementary Table 2 provides the RNA sequencing data of Panc-1 cells transfected with scrambled shRNAs and TACC3 shRNAs. Supplementary Table 3 provides protein information that interacted with TACC3 obtained by the Co-IP experiment combined with mass spectrometry. Supplementary Table 4 provides the clinical data of 54 patients who received neoadjuvant therapy in our study. Supplementary Table 5 provides the RNA sequencing data of gemcitabine-resistant and gemcitabine-sensitive Panc-1 cell lines. Original data files provide the original western blots. The online databases used in our study including The Cancer Genome Atlas (TCGA, https://portal.gdc.cancer.gov/); Genotype-Tissue Expression Project (GTEx, https://www.genome.gov/Funded-Programs-Projects/Genotype-Tissue-Expression-Project); Protein-Protein Interaction Network (PPI Network, https://fgrtools.hms.harvard.edu/MIST); Gene Expression Profiling Interactive Analysis (GEPIA, http://gepia2.cancer-pku.cn/). The data supporting the findings of this study are available within the article and its supplementary files.
