# Supplementary material for: Knockdown of TACC3 inhibits tumor cell proliferation and increases chemosensitivity in pancreatic cancer
Source: Cell Death Dis. 2023 Nov 27;14(11):778. doi: 10.1038/s41419-023-06313-x (PMC10682013; doi:10.1038/s41419-023-06313-x)
Supplement: Supplementary file 1 — Supplementary Material Legends [file 41419_2023_6313_MOESM1_ESM.doc]

**Supplementary Material Legends for**

**Knockdown of TACC3 inhibits tumor cell proliferation and increases chemosensitivity in pancreatic cancer**

Saimeng Shi^1,2,3,4,#^, Duancheng Guo^1,2,3,4,#^, Longyun Ye^1,2,3,4,#^, Tianjiao Li^1,2,3,4^, Qinglin Fei^1,2,3,4^, Mengxiong Lin^1,2,3,4^, Xianjun Yu ^1,2,3,4,*^, Kaizhou Jin ^1,2,3,4,*^, Weiding Wu^1,2,3,4,*^

^1^Department of Pancreatic Surgery, Fudan University Shanghai Cancer Center, Shanghai 200032, China;

^2^Department of Oncology, Shanghai Medical College, Fudan University, Shanghai 200032, China;

^3^Shanghai Pancreatic Cancer Institute, Shanghai 200032, China;

^4^Pancreatic Cancer Institute, Fudan University, Shanghai 200032, China;

^*^ Correspondence to: wuweiding@fudanpci.org (Weiding Wu),

jinkaizhou@fudanpci.org (Kaizhou Jin) or

yuxianjun@fudanpci.org (Xianjun Yu).

^#^These authors contributed equally to this work.

**This file includes Captions for Supplementary Tables 1 to 6.**

**Captions for Supplementary Tables 1 to 6**

**Supplementary Table 1****:** Clinical data of 218 patients included in the tissue microarray.

**Supplementary Table 2:** RNA sequencing data of Panc-1 cells transfected with scrambled shRNAs and TACC3 shRNAs.

**Supplementary Table 3:** Protein information that interacted with TACC3 obtained by the Co-IP experiment combined with mass spectrometry.

**Supplementary Table 4:** Clinical data of 54 patients who received neoadjuvant therapy in our study.

**Supplementary Table 5:** RNA sequencing data of gemcitabine-resistant and gemcitabine-sensitive Panc-1 cell lines.

**Original data files:** Original western blots.
