# Supplementary figures and images for: Knockdown of TACC3 inhibits tumor cell proliferation and increases chemosensitivity in pancreatic cancer
Source: Cell Death Dis. 2023 Nov 27;14(11):778. doi: 10.1038/s41419-023-06313-x (PMC10682013; doi:10.1038/s41419-023-06313-x)

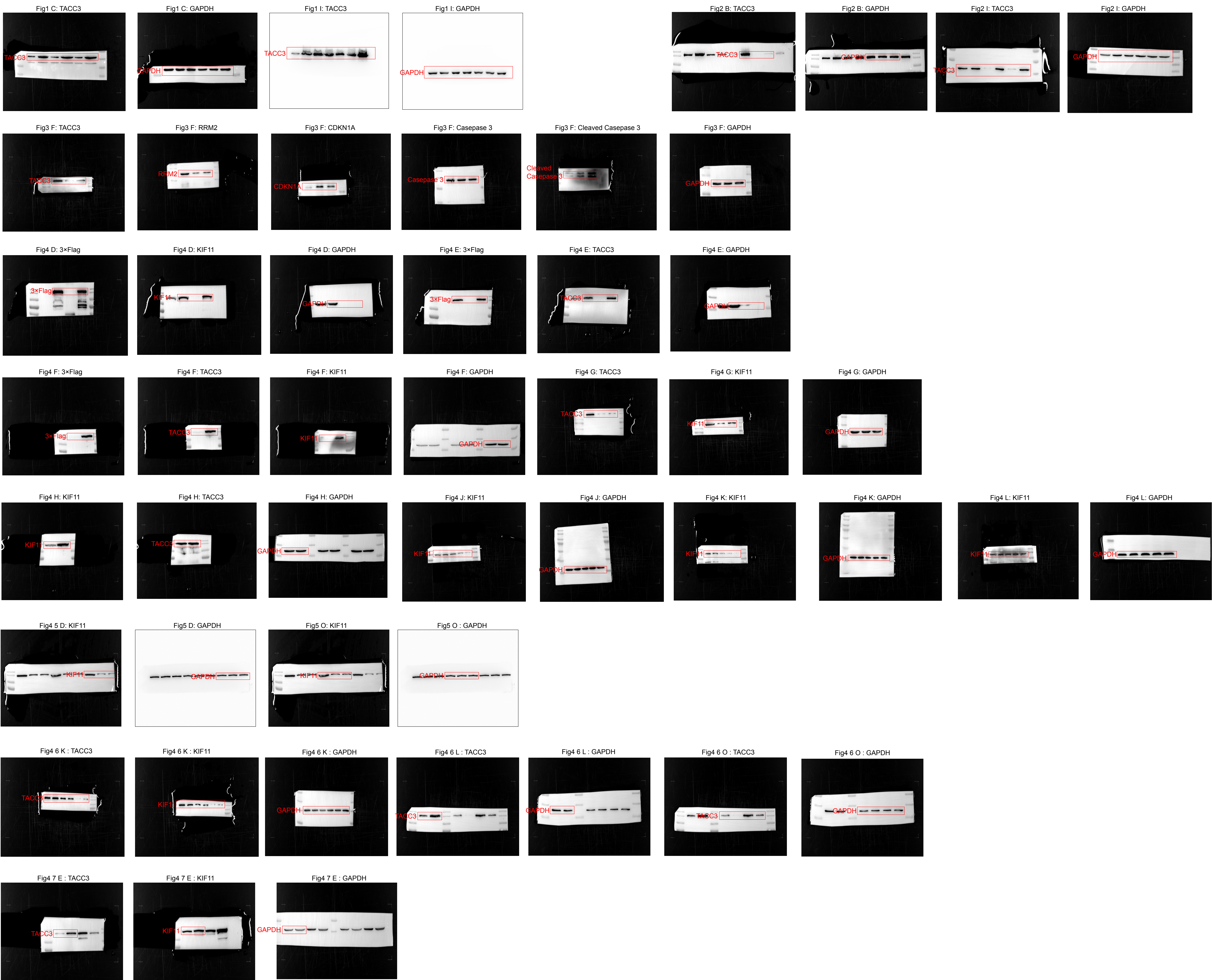

Supplement: Supplementary file 7 — original data files [file 41419_2023_6313_MOESM7_ESM.pdf]
